# 7-Chloro-4-(piperazin-1-yl)quinoline

**DOI:** 10.1107/S1600536812014912

**Published:** 2012-04-21

**Authors:** Amol A. Kulkarni, Christopher L. King, Joseph M. D. Fortunak, Ray J. Butcher

**Affiliations:** a College of Pharmacy, Howard University, 2300 4th Street, NW, Washington, DC 2059, USA; b Department of Chemistry, Howard University, 525 College Street, NW, Washington, DC 2059, USA

## Abstract

There are two mol­ecules in the asymmetric unit (*Z*′ = 2) of the title compound, C_13_H_14_ClN_3_, Each mol­ecule is linked by N—H⋯N hydrogen bonds to another of the same type in a chain in [110]. The crystal studied was a non-merohedral twin with components 0.622 (2) and 0.378 (2).

## Related literature

The title compound is an important inter­mediate in the synthesis of the anti-malarial compound piperaquine {systematic name: 7-chloro-4-[4-[3-[4-(7-chloro­quinolin-4-yl)piperazin-1-yl]prop­yl]piperazin-1-yl]quinoline phospho­ric acid}, see: Chen *et al.* (1982 ▶); Hien *et al.* (2004 ▶); Dongre *et al.* (2007 ▶).
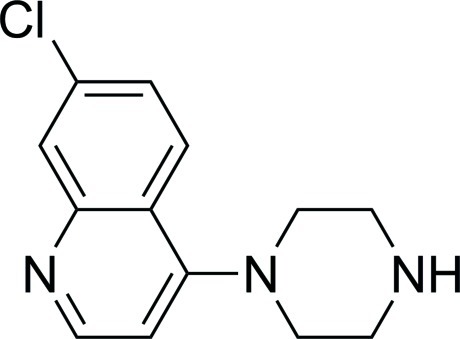


## Experimental

### 

#### Crystal data

C_13_H_14_ClN_3_
*M* *_r_* = 247.72
Triclinic, 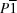

*a* = 7.0048 (6) Å
*b* = 7.8297 (8) Å
*c* = 21.4256 (19) Å
α = 91.371 (8)°
β = 91.292 (7)°
γ = 95.210 (8)°
*V* = 1169.55 (19) Å^3^
*Z* = 4
Cu *K*α radiation
μ = 2.72 mm^−1^
*T* = 123 K
0.43 × 0.35 × 0.12 mm

#### Data collection

Oxford Diffraction Xcalibur Ruby Gemini diffractometer
Absorption correction: multi-scan (*CrysAlis PRO*; Oxford Diffraction, 2007 ▶) *T*_min_ = 0.809, *T*_max_ = 1.000
6990 measured reflections
6990 independent reflections
5619 reflections with *I* > 2σ(*I*)
*R*_int_ = 0.000

#### Refinement

*R*[*F* ^2^ > 2σ(*F* ^2^)] = 0.074
*wR*(*F* ^2^) = 0.228
*S* = 1.09
6990 reflections
316 parameters
H atoms treated by a mixture of independent and constrained refinement
Δρ_max_ = 0.65 e Å^−3^
Δρ_min_ = −0.60 e Å^−3^

### 

Data collection: *CrysAlis PRO* (Oxford Diffraction, 2007 ▶); cell refinement: *CrysAlis PRO*; data reduction: *CrysAlis PRO*; program(s) used to solve structure: *SHELXS97* (Sheldrick, 2008 ▶); program(s) used to refine structure: *SHELXL97* (Sheldrick, 2008 ▶); molecular graphics: *SHELXTL* (Sheldrick, 2008 ▶); software used to prepare material for publication: *SHELXTL*.

## Supplementary Material

Crystal structure: contains datablock(s) I, global. DOI: 10.1107/S1600536812014912/bt5836sup1.cif

Structure factors: contains datablock(s) I. DOI: 10.1107/S1600536812014912/bt5836Isup2.hkl

Supplementary material file. DOI: 10.1107/S1600536812014912/bt5836Isup3.cml

Additional supplementary materials:  crystallographic information; 3D view; checkCIF report

## Figures and Tables

**Table 1:** Hydrogen-bond geometry (Å, °)

*D*—H⋯*A*	*D*—H	H⋯*A*	*D*⋯*A*	*D*—H⋯*A*
N3*A*—H3*AN*⋯N1*A*^i^	0.92 (4)	2.18 (4)	3.083 (4)	166 (4)
N3*B*—H3*BN*⋯N1*B*^i^	0.99 (4)	2.12 (4)	3.088 (4)	166 (4)

Symmetry code: (i) 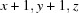.

**Fig. 1.**
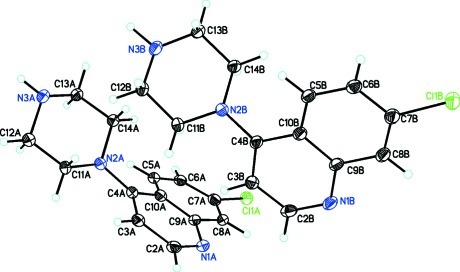
A view of the title compound, C13H14ClN3, showing atom numbering scheme and the two molecules in the asymmetric unit.

**Fig. 2.**
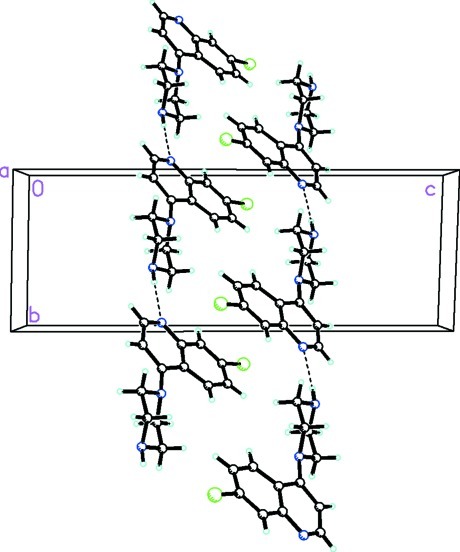
A view of the packing of the molecules showing the chains of molecules linked by N—H···N hydrogen bonds (shown by dashed lines).

## supplementary crystallographic information

### Comment

Recrystallization of the title compound from 2-propanol removes low levels (1–4%) of impurities that are present from the manufacturing process. Impurities in the desired product arise from the presence of 4,5-dichloroquinoline in 4,7-DCQ and are difficult to remove from the manufacturing process of commercial malaria drugs, including amodiaquine and piperaquine (Dongre *et al.*, 2007).

In view of the pharmaceutical importance of this intermediate its crystal structure was determined. There are two molecules in the asymmetric unit (*Z*' = 2). Each molecule is linked by N—H···N hydrogen bonds to another of the same type in a chain in the b direction.

### Experimental

A solution of 4,7-dichloroquinoline (10 g, 51 mmole, 1 equiv) and piperazine (13.05 g, 153 mmole, 3 equiv) in 2-propanol (25 ml) was heated to a gentle reflux for 4 h. The solution was cooled to room temperature. Ethyl acetate (50 ml) was added and the reaction mixture was stirred at room temperature for 14 h. It was then poured into a separatory funnel and was washed with water (3 *X* 50 ml). The organic layer was dried using anhydrous Na_2_SO_4_. Removal of the solvent *in vacuo* resulted in the isolation of the desired compound as pale yellow crystals. The crude product was recrystallized from 2-propanol to yield colorless crystals of the desired compound. mp 112–114 °C; ^1^H-NMR (CDCl_3_) d 8.68 (d, J = 4.8 Hz, 1H), 8. 01 (d, J = 9.2 Hz, 1H), 7.69 (d, J = 2.4 Hz, 1H), 7.55 (dd, J = 9.2, 2.4 Hz, 1H), 6.96 (d, J = 4.8 Hz, 1H), 3.12–2.93 (m, 8H).

### Refinement

H atoms were placed in geometrically idealized positions and constrained to ride on their parent atoms with a C—H distance of 0.95 and 0.99 [*U*_iso_(H) = 1.2*U*_eq_(C)]. The H atoms attached to N were refined isotropically. The structure was a non-merohedral twin with components 0.622 (2) and 0.378 (2).

### Crystal data

**Table:**

C_13_H_14_ClN_3_	*Z* = 4
*M**_r_* = 247.72	*F*(000) = 520
Triclinic, *P*1	*D*_x_ = 1.407 Mg m^−^^3^
*a* = 7.0048 (6) Å	Cu *K*α radiation, λ = 1.54184 Å
*b* = 7.8297 (8) Å	Cell parameters from 1440 reflections
*c* = 21.4256 (19) Å	θ = 4.1–75.3°
α = 91.371 (8)°	µ = 2.72 mm^−^^1^
β = 91.292 (7)°	*T* = 123 K
γ = 95.210 (8)°	Triangular plate, colorless
*V* = 1169.55 (19) Å^3^	0.43 × 0.35 × 0.12 mm

### Data collection

**Table:**

Oxford Diffraction Xcalibur Ruby Gemini diffractometer	6990 independent reflections
Radiation source: fine-focus sealed tube	5619 reflections with *I* > 2σ(*I*)
Graphite monochromator	*R*_int_ = 0.000
Detector resolution: 10.5081 pixels mm^-1^	θ_max_ = 75.9°, θ_min_ = 4.1°
ω scans	*h* = −8→8
Absorption correction: multi-scan (*CrysAlis PRO*; Oxford Diffraction, 2007)	*k* = −9→9
*T*_min_ = 0.809, *T*_max_ = 1.000	*l* = −20→26
6990 measured reflections	

### Refinement

**Table:**

Refinement on *F*^2^	Primary atom site location: structure-invariant direct methods
Least-squares matrix: full	Secondary atom site location: difference Fourier map
*R*[*F*^2^ > 2σ(*F*^2^)] = 0.074	Hydrogen site location: inferred from neighbouring sites
*wR*(*F*^2^) = 0.228	H atoms treated by a mixture of independent and constrained refinement
*S* = 1.09	*w* = 1/[σ^2^(*F*_o_^2^) + (0.1441*P*)^2^ + 0.6728*P*] where *P* = (*F*_o_^2^ + 2*F*_c_^2^)/3
6990 reflections	(Δ/σ)_max_ = 0.001
316 parameters	Δρ_max_ = 0.65 e Å^−^^3^
0 restraints	Δρ_min_ = −0.60 e Å^−^^3^

### Special details

**Table:**

Geometry. All e.s.d.'s (except the e.s.d. in the dihedral angle between two l.s. planes) are estimated using the full covariance matrix. The cell e.s.d.'s are taken into account individually in the estimation of e.s.d.'s in distances, angles and torsion angles; correlations between e.s.d.'s in cell parameters are only used when they are defined by crystal symmetry. An approximate (isotropic) treatment of cell e.s.d.'s is used for estimating e.s.d.'s involving l.s. planes.
Refinement. Refinement of *F*^2^ against ALL reflections. The weighted *R*-factor *wR* and goodness of fit *S* are based on *F*^2^, conventional *R*-factors *R* are based on *F*, with *F* set to zero for negative *F*^2^. The threshold expression of *F*^2^ > σ(*F*^2^) is used only for calculating *R*-factors(gt) *etc*. and is not relevant to the choice of reflections for refinement. *R*-factors based on *F*^2^ are statistically about twice as large as those based on *F*, and *R*- factors based on ALL data will be even larger.

### Fractional atomic coordinates and isotropic or equivalent isotropic displacement parameters (Å^2^)

**Table:**

	*x*	*y*	*z*	*U*_iso_*/*U*_eq_	
Cl1A	0.17772 (13)	0.19345 (12)	0.52611 (4)	0.0394 (3)	
N1A	0.5120 (4)	−0.0737 (4)	0.34594 (14)	0.0319 (6)	
N2A	0.9733 (4)	0.3190 (4)	0.35392 (14)	0.0289 (6)	
N3A	1.2344 (5)	0.6024 (4)	0.32035 (15)	0.0343 (7)	
H3AN	1.333 (6)	0.688 (5)	0.3255 (18)	0.023 (9)*	
C2A	0.6631 (6)	−0.0790 (5)	0.31036 (17)	0.0324 (7)	
H2AA	0.6654	−0.1744	0.2822	0.039*	
C3A	0.8204 (5)	0.0455 (5)	0.31094 (17)	0.0309 (7)	
H3AA	0.9209	0.0349	0.2825	0.037*	
C4A	0.8298 (5)	0.1837 (4)	0.35280 (16)	0.0276 (7)	
C5A	0.6879 (5)	0.2915 (4)	0.45125 (16)	0.0302 (7)	
H5AA	0.7997	0.3675	0.4595	0.036*	
C6A	0.5383 (5)	0.2906 (4)	0.49154 (16)	0.0303 (7)	
H6AA	0.5461	0.3641	0.5276	0.036*	
C7A	0.3736 (5)	0.1788 (5)	0.47835 (17)	0.0328 (7)	
C8A	0.3637 (5)	0.0620 (4)	0.43035 (17)	0.0309 (7)	
H8AA	0.2523	−0.0154	0.4235	0.037*	
C9A	0.5227 (5)	0.0570 (4)	0.39031 (16)	0.0285 (7)	
C10A	0.6789 (5)	0.1820 (4)	0.39786 (16)	0.0285 (7)	
C11A	1.1413 (5)	0.2939 (5)	0.31546 (17)	0.0301 (7)	
H11A	1.1048	0.2980	0.2706	0.036*	
H11B	1.1863	0.1799	0.3234	0.036*	
C12A	1.3017 (5)	0.4336 (5)	0.33146 (17)	0.0326 (7)	
H12A	1.3421	0.4263	0.3758	0.039*	
H12B	1.4137	0.4173	0.3053	0.039*	
C13A	1.0730 (5)	0.6282 (5)	0.36040 (18)	0.0321 (7)	
H13A	1.0295	0.7433	0.3537	0.038*	
H13B	1.1139	0.6219	0.4048	0.038*	
C14A	0.9098 (5)	0.4920 (4)	0.34551 (17)	0.0300 (7)	
H14A	0.8018	0.5088	0.3734	0.036*	
H14B	0.8641	0.5029	0.3019	0.036*	
Cl1B	−0.32087 (13)	0.18514 (12)	−0.02405 (4)	0.0397 (2)	
N1B	0.0489 (4)	−0.0420 (4)	0.15733 (15)	0.0335 (6)	
N2B	0.5041 (4)	0.3518 (4)	0.14536 (14)	0.0279 (6)	
N3B	0.7621 (4)	0.6426 (4)	0.17856 (15)	0.0334 (7)	
H3BN	0.870 (6)	0.730 (5)	0.1712 (18)	0.024 (10)*	
C2B	0.2060 (6)	−0.0387 (5)	0.19218 (17)	0.0335 (8)	
H2BA	0.2141	−0.1278	0.2212	0.040*	
C3B	0.3630 (5)	0.0859 (5)	0.19001 (17)	0.0307 (7)	
H3BA	0.4700	0.0811	0.2178	0.037*	
C4B	0.3616 (5)	0.2153 (4)	0.14743 (16)	0.0267 (7)	
C5B	0.2016 (5)	0.3037 (4)	0.04913 (16)	0.0289 (7)	
H5BA	0.3105	0.3800	0.0403	0.035*	
C6B	0.0448 (5)	0.2931 (4)	0.00929 (16)	0.0293 (7)	
H6BA	0.0455	0.3597	−0.0272	0.035*	
C7B	−0.1172 (5)	0.1822 (5)	0.02324 (17)	0.0325 (7)	
C8B	−0.1169 (5)	0.0742 (4)	0.07227 (17)	0.0315 (7)	
H8BA	−0.2261	−0.0029	0.0799	0.038*	
C9B	0.0492 (5)	0.0790 (4)	0.11170 (17)	0.0286 (7)	
C10B	0.2044 (5)	0.2034 (4)	0.10308 (16)	0.0281 (7)	
C11B	0.6787 (5)	0.3355 (5)	0.18261 (16)	0.0293 (7)	
H11C	0.7254	0.2218	0.1742	0.035*	
H11D	0.6506	0.3445	0.2276	0.035*	
C12B	0.8319 (5)	0.4759 (5)	0.16633 (17)	0.0313 (7)	
H12C	0.9498	0.4652	0.1917	0.038*	
H12D	0.8635	0.4645	0.1217	0.038*	
C13B	0.5927 (5)	0.6607 (5)	0.13965 (18)	0.0320 (7)	
H13C	0.6250	0.6497	0.0951	0.038*	
H13D	0.5483	0.7759	0.1469	0.038*	
C14B	0.4344 (5)	0.5242 (4)	0.15480 (17)	0.0297 (7)	
H14C	0.3963	0.5394	0.1987	0.036*	
H14D	0.3208	0.5349	0.1273	0.036*	

### Atomic displacement parameters (Å^2^)

**Table:**

	*U*^11^	*U*^22^	*U*^33^	*U*^12^	*U*^13^	*U*^23^
Cl1A	0.0319 (5)	0.0398 (5)	0.0473 (5)	0.0056 (4)	0.0108 (4)	0.0045 (4)
N1A	0.0303 (15)	0.0247 (14)	0.0404 (15)	0.0006 (12)	−0.0019 (12)	0.0017 (11)
N2A	0.0248 (14)	0.0273 (15)	0.0352 (14)	0.0050 (12)	0.0037 (11)	0.0018 (11)
N3A	0.0282 (15)	0.0297 (16)	0.0448 (17)	−0.0010 (12)	0.0058 (12)	0.0033 (12)
C2A	0.0365 (19)	0.0235 (16)	0.0376 (18)	0.0060 (14)	−0.0009 (14)	−0.0025 (13)
C3A	0.0276 (17)	0.0276 (17)	0.0385 (18)	0.0066 (14)	0.0045 (13)	0.0017 (13)
C4A	0.0231 (16)	0.0255 (16)	0.0345 (17)	0.0038 (13)	−0.0008 (12)	0.0046 (12)
C5A	0.0276 (17)	0.0252 (16)	0.0380 (17)	0.0027 (13)	−0.0003 (13)	0.0040 (13)
C6A	0.0339 (18)	0.0240 (15)	0.0338 (16)	0.0064 (13)	0.0031 (13)	0.0017 (12)
C7A	0.0319 (18)	0.0311 (18)	0.0368 (17)	0.0080 (14)	0.0061 (14)	0.0054 (13)
C8A	0.0250 (16)	0.0244 (16)	0.0431 (18)	0.0010 (12)	0.0003 (13)	0.0054 (13)
C9A	0.0291 (17)	0.0210 (15)	0.0351 (17)	0.0012 (13)	−0.0034 (13)	0.0026 (12)
C10A	0.0266 (17)	0.0266 (17)	0.0332 (16)	0.0064 (14)	0.0019 (13)	0.0027 (13)
C11A	0.0207 (15)	0.0298 (17)	0.0403 (18)	0.0038 (13)	0.0045 (13)	0.0031 (13)
C12A	0.0284 (18)	0.0316 (18)	0.0383 (17)	0.0045 (14)	0.0021 (14)	0.0039 (14)
C13A	0.0276 (17)	0.0252 (17)	0.0430 (18)	0.0004 (13)	0.0022 (14)	−0.0005 (13)
C14A	0.0255 (16)	0.0256 (16)	0.0391 (17)	0.0024 (13)	0.0025 (13)	0.0022 (13)
Cl1B	0.0304 (5)	0.0384 (5)	0.0496 (5)	0.0016 (4)	−0.0044 (3)	−0.0019 (4)
N1B	0.0287 (15)	0.0258 (14)	0.0453 (17)	−0.0038 (11)	0.0085 (12)	0.0034 (12)
N2B	0.0228 (13)	0.0243 (14)	0.0368 (15)	0.0037 (11)	0.0031 (11)	0.0004 (11)
N3B	0.0272 (15)	0.0296 (15)	0.0425 (16)	−0.0039 (12)	0.0043 (12)	0.0014 (12)
C2B	0.038 (2)	0.0230 (16)	0.0394 (19)	0.0018 (15)	0.0087 (15)	0.0044 (13)
C3B	0.0264 (17)	0.0277 (16)	0.0384 (18)	0.0025 (14)	0.0043 (13)	0.0038 (13)
C4B	0.0220 (15)	0.0225 (16)	0.0356 (17)	0.0012 (13)	0.0063 (13)	−0.0004 (12)
C5B	0.0251 (16)	0.0205 (14)	0.0407 (17)	−0.0001 (12)	0.0057 (13)	−0.0011 (12)
C6B	0.0305 (17)	0.0232 (15)	0.0339 (16)	0.0015 (13)	0.0037 (13)	−0.0017 (12)
C7B	0.0286 (17)	0.0282 (17)	0.0404 (18)	0.0026 (14)	0.0018 (14)	−0.0026 (14)
C8B	0.0243 (16)	0.0245 (16)	0.0454 (18)	−0.0009 (12)	0.0070 (13)	−0.0027 (13)
C9B	0.0258 (16)	0.0198 (15)	0.0400 (18)	−0.0004 (13)	0.0072 (13)	−0.0006 (13)
C10B	0.0244 (16)	0.0226 (16)	0.0373 (17)	0.0019 (13)	0.0057 (13)	−0.0008 (12)
C11B	0.0214 (16)	0.0291 (17)	0.0377 (17)	0.0041 (13)	0.0023 (13)	0.0022 (13)
C12B	0.0224 (16)	0.0304 (17)	0.0411 (18)	0.0015 (13)	0.0053 (13)	0.0036 (14)
C13B	0.0277 (17)	0.0248 (16)	0.0431 (18)	−0.0018 (13)	0.0041 (14)	0.0036 (13)
C14B	0.0263 (16)	0.0221 (16)	0.0406 (18)	0.0023 (13)	0.0028 (13)	0.0002 (13)

### Geometric parameters (Å, º)

**Table:**

Cl1A—C7A	1.740 (4)	Cl1B—C7B	1.733 (4)
N1A—C2A	1.321 (5)	N1B—C2B	1.314 (5)
N1A—C9A	1.376 (5)	N1B—C9B	1.378 (5)
N2A—C4A	1.392 (4)	N2B—C4B	1.396 (4)
N2A—C11A	1.477 (4)	N2B—C11B	1.462 (4)
N2A—C14A	1.477 (4)	N2B—C14B	1.488 (4)
N3A—C13A	1.460 (5)	N3B—C13B	1.454 (5)
N3A—C12A	1.466 (5)	N3B—C12B	1.455 (5)
N3A—H3AN	0.92 (4)	N3B—H3BN	0.99 (4)
C2A—C3A	1.402 (5)	C2B—C3B	1.405 (5)
C2A—H2AA	0.9500	C2B—H2BA	0.9500
C3A—C4A	1.385 (5)	C3B—C4B	1.380 (5)
C3A—H3AA	0.9500	C3B—H3BA	0.9500
C4A—C10A	1.447 (5)	C4B—C10B	1.433 (5)
C5A—C6A	1.372 (5)	C5B—C6B	1.371 (5)
C5A—C10A	1.410 (5)	C5B—C10B	1.414 (5)
C5A—H5AA	0.9500	C5B—H5BA	0.9500
C6A—C7A	1.402 (5)	C6B—C7B	1.408 (5)
C6A—H6AA	0.9500	C6B—H6BA	0.9500
C7A—C8A	1.356 (5)	C7B—C8B	1.364 (5)
C8A—C9A	1.424 (5)	C8B—C9B	1.419 (5)
C8A—H8AA	0.9500	C8B—H8BA	0.9500
C9A—C10A	1.404 (4)	C9B—C10B	1.411 (4)
C11A—C12A	1.523 (5)	C11B—C12B	1.518 (4)
C11A—H11A	0.9900	C11B—H11C	0.9900
C11A—H11B	0.9900	C11B—H11D	0.9900
C12A—H12A	0.9900	C12B—H12C	0.9900
C12A—H12B	0.9900	C12B—H12D	0.9900
C13A—C14A	1.514 (5)	C13B—C14B	1.515 (4)
C13A—H13A	0.9900	C13B—H13C	0.9900
C13A—H13B	0.9900	C13B—H13D	0.9900
C14A—H14A	0.9900	C14B—H14C	0.9900
C14A—H14B	0.9900	C14B—H14D	0.9900
			
C2A—N1A—C9A	115.7 (3)	C2B—N1B—C9B	115.9 (3)
C4A—N2A—C11A	115.9 (3)	C4B—N2B—C11B	116.3 (3)
C4A—N2A—C14A	116.3 (3)	C4B—N2B—C14B	114.5 (3)
C11A—N2A—C14A	110.7 (3)	C11B—N2B—C14B	111.2 (3)
C13A—N3A—C12A	109.6 (3)	C13B—N3B—C12B	109.8 (3)
C13A—N3A—H3AN	113 (3)	C13B—N3B—H3BN	114 (2)
C12A—N3A—H3AN	111 (3)	C12B—N3B—H3BN	107 (2)
N1A—C2A—C3A	125.0 (3)	N1B—C2B—C3B	125.3 (3)
N1A—C2A—H2AA	117.5	N1B—C2B—H2BA	117.4
C3A—C2A—H2AA	117.5	C3B—C2B—H2BA	117.4
C4A—C3A—C2A	120.3 (3)	C4B—C3B—C2B	119.8 (3)
C4A—C3A—H3AA	119.8	C4B—C3B—H3BA	120.1
C2A—C3A—H3AA	119.8	C2B—C3B—H3BA	120.1
C3A—C4A—N2A	124.3 (3)	C3B—C4B—N2B	123.6 (3)
C3A—C4A—C10A	116.1 (3)	C3B—C4B—C10B	116.5 (3)
N2A—C4A—C10A	119.6 (3)	N2B—C4B—C10B	119.9 (3)
C6A—C5A—C10A	121.3 (3)	C6B—C5B—C10B	121.2 (3)
C6A—C5A—H5AA	119.3	C6B—C5B—H5BA	119.4
C10A—C5A—H5AA	119.3	C10B—C5B—H5BA	119.4
C5A—C6A—C7A	118.6 (3)	C5B—C6B—C7B	119.1 (3)
C5A—C6A—H6AA	120.7	C5B—C6B—H6BA	120.5
C7A—C6A—H6AA	120.7	C7B—C6B—H6BA	120.5
C8A—C7A—C6A	122.2 (3)	C8B—C7B—C6B	121.9 (3)
C8A—C7A—Cl1A	120.0 (3)	C8B—C7B—Cl1B	120.1 (3)
C6A—C7A—Cl1A	117.8 (3)	C6B—C7B—Cl1B	118.1 (3)
C7A—C8A—C9A	119.1 (3)	C7B—C8B—C9B	118.9 (3)
C7A—C8A—H8AA	120.5	C7B—C8B—H8BA	120.5
C9A—C8A—H8AA	120.5	C9B—C8B—H8BA	120.5
N1A—C9A—C10A	124.0 (3)	N1B—C9B—C10B	123.1 (3)
N1A—C9A—C8A	116.5 (3)	N1B—C9B—C8B	116.8 (3)
C10A—C9A—C8A	119.5 (3)	C10B—C9B—C8B	120.1 (3)
C9A—C10A—C5A	118.5 (3)	C9B—C10B—C5B	118.1 (3)
C9A—C10A—C4A	118.2 (3)	C9B—C10B—C4B	118.6 (3)
C5A—C10A—C4A	123.1 (3)	C5B—C10B—C4B	123.2 (3)
N2A—C11A—C12A	109.8 (3)	N2B—C11B—C12B	109.8 (3)
N2A—C11A—H11A	109.7	N2B—C11B—H11C	109.7
C12A—C11A—H11A	109.7	C12B—C11B—H11C	109.7
N2A—C11A—H11B	109.7	N2B—C11B—H11D	109.7
C12A—C11A—H11B	109.7	C12B—C11B—H11D	109.7
H11A—C11A—H11B	108.2	H11C—C11B—H11D	108.2
N3A—C12A—C11A	109.7 (3)	N3B—C12B—C11B	109.4 (3)
N3A—C12A—H12A	109.7	N3B—C12B—H12C	109.8
C11A—C12A—H12A	109.7	C11B—C12B—H12C	109.8
N3A—C12A—H12B	109.7	N3B—C12B—H12D	109.8
C11A—C12A—H12B	109.7	C11B—C12B—H12D	109.8
H12A—C12A—H12B	108.2	H12C—C12B—H12D	108.2
N3A—C13A—C14A	109.9 (3)	N3B—C13B—C14B	110.1 (3)
N3A—C13A—H13A	109.7	N3B—C13B—H13C	109.6
C14A—C13A—H13A	109.7	C14B—C13B—H13C	109.6
N3A—C13A—H13B	109.7	N3B—C13B—H13D	109.6
C14A—C13A—H13B	109.7	C14B—C13B—H13D	109.6
H13A—C13A—H13B	108.2	H13C—C13B—H13D	108.1
N2A—C14A—C13A	110.5 (3)	N2B—C14B—C13B	109.3 (3)
N2A—C14A—H14A	109.6	N2B—C14B—H14C	109.8
C13A—C14A—H14A	109.6	C13B—C14B—H14C	109.8
N2A—C14A—H14B	109.6	N2B—C14B—H14D	109.8
C13A—C14A—H14B	109.6	C13B—C14B—H14D	109.8
H14A—C14A—H14B	108.1	H14C—C14B—H14D	108.3
			
C9A—N1A—C2A—C3A	−6.3 (5)	C9B—N1B—C2B—C3B	6.0 (5)
N1A—C2A—C3A—C4A	2.9 (5)	N1B—C2B—C3B—C4B	−2.0 (5)
C2A—C3A—C4A—N2A	−175.4 (3)	C2B—C3B—C4B—N2B	174.7 (3)
C2A—C3A—C4A—C10A	5.0 (5)	C2B—C3B—C4B—C10B	−6.2 (5)
C11A—N2A—C4A—C3A	−11.6 (4)	C11B—N2B—C4B—C3B	11.9 (4)
C14A—N2A—C4A—C3A	121.2 (4)	C14B—N2B—C4B—C3B	−120.0 (4)
C11A—N2A—C4A—C10A	167.9 (3)	C11B—N2B—C4B—C10B	−167.1 (3)
C14A—N2A—C4A—C10A	−59.3 (4)	C14B—N2B—C4B—C10B	60.9 (4)
C10A—C5A—C6A—C7A	−0.6 (5)	C10B—C5B—C6B—C7B	1.1 (5)
C5A—C6A—C7A—C8A	5.8 (5)	C5B—C6B—C7B—C8B	−5.7 (5)
C5A—C6A—C7A—Cl1A	−174.4 (3)	C5B—C6B—C7B—Cl1B	174.4 (3)
C6A—C7A—C8A—C9A	−2.7 (5)	C6B—C7B—C8B—C9B	2.5 (5)
Cl1A—C7A—C8A—C9A	177.4 (3)	Cl1B—C7B—C8B—C9B	−177.6 (3)
C2A—N1A—C9A—C10A	1.6 (5)	C2B—N1B—C9B—C10B	−1.8 (5)
C2A—N1A—C9A—C8A	−178.2 (3)	C2B—N1B—C9B—C8B	178.1 (3)
C7A—C8A—C9A—N1A	174.3 (3)	C7B—C8B—C9B—N1B	−174.7 (3)
C7A—C8A—C9A—C10A	−5.5 (5)	C7B—C8B—C9B—C10B	5.2 (5)
N1A—C9A—C10A—C5A	−169.5 (3)	N1B—C9B—C10B—C5B	170.4 (3)
C8A—C9A—C10A—C5A	10.4 (5)	C8B—C9B—C10B—C5B	−9.4 (5)
N1A—C9A—C10A—C4A	6.0 (5)	N1B—C9B—C10B—C4B	−6.2 (5)
C8A—C9A—C10A—C4A	−174.1 (3)	C8B—C9B—C10B—C4B	174.0 (3)
C6A—C5A—C10A—C9A	−7.4 (5)	C6B—C5B—C10B—C9B	6.3 (5)
C6A—C5A—C10A—C4A	177.4 (3)	C6B—C5B—C10B—C4B	−177.3 (3)
C3A—C4A—C10A—C9A	−9.0 (5)	C3B—C4B—C10B—C9B	9.8 (5)
N2A—C4A—C10A—C9A	171.4 (3)	N2B—C4B—C10B—C9B	−171.0 (3)
C3A—C4A—C10A—C5A	166.3 (3)	C3B—C4B—C10B—C5B	−166.6 (3)
N2A—C4A—C10A—C5A	−13.3 (5)	N2B—C4B—C10B—C5B	12.6 (5)
C4A—N2A—C11A—C12A	−168.4 (3)	C4B—N2B—C11B—C12B	169.6 (3)
C14A—N2A—C11A—C12A	56.3 (4)	C14B—N2B—C11B—C12B	−56.9 (4)
C13A—N3A—C12A—C11A	61.2 (4)	C13B—N3B—C12B—C11B	−61.4 (4)
N2A—C11A—C12A—N3A	−58.8 (4)	N2B—C11B—C12B—N3B	59.1 (4)
C12A—N3A—C13A—C14A	−60.7 (4)	C12B—N3B—C13B—C14B	61.3 (4)
C4A—N2A—C14A—C13A	168.8 (3)	C4B—N2B—C14B—C13B	−169.5 (3)
C11A—N2A—C14A—C13A	−56.1 (4)	C11B—N2B—C14B—C13B	56.1 (4)
N3A—C13A—C14A—N2A	58.1 (4)	N3B—C13B—C14B—N2B	−57.8 (4)

### Hydrogen-bond geometry (Å, º)

**Table:**

*D*—H···*A*	*D*—H	H···*A*	*D*···*A*	*D*—H···*A*
N3*A*—H3*AN*···N1*A*^i^	0.92 (4)	2.18 (4)	3.083 (4)	166 (4)
N3*B*—H3*BN*···N1*B*^i^	0.99 (4)	2.12 (4)	3.088 (4)	166 (4)

Symmetry code: (i) *x*+1, *y*+1, *z*.
